# Supplementary material for: MetaRibo-Seq measures translation in microbiomes
Source: Nat Commun. 2020 Jun 29;11:3268. doi: 10.1038/s41467-020-17081-z (PMC7324362; doi:10.1038/s41467-020-17081-z)
Supplement: Supplementary file 10 — Supplementary Data 7 [file 41467_2020_17081_MOESM10_ESM.zip › File2/Confidence_High_Taxonomy/377741_out.krona.html]

Javascript must be enabled to view this page.

members
magnitude
magnitudeUnassigned
count
unassigned
taxon
rank

377741\_out

19

2
19
superkingdom

phylum
19
1239

class
19
186801

186802
19
order

19
186803
family

genus
19
841

species

SRS011405\_contig\_number\_16868SRS013951\_contig\_number\_40522SRS014459\_contig\_number\_6657SRS014923\_contig\_number\_28037SRS016335\_contig\_number\_40195SRS017433\_contig\_number\_contig-100\_459.110268SRS019161\_contig\_number\_34862SRS019445\_contig\_number\_contig-100\_88.205652SRS048164\_contig\_number\_31494SRS049959\_contig\_number\_41969SRS063040\_contig\_number\_46247SRS064276\_contig\_number\_40927SRS143085\_contig\_number\_5152SRS143417\_contig\_number\_4468SRS143722\_contig\_number\_16668SRS147271\_contig\_number\_40699SRS147977\_contig\_number\_5671SRS893288\_contig\_number\_1236SRS893327\_contig\_number\_10363
19
360807
